# MicroRNAs: fundamental regulators of gene expression in major affective disorders and suicidal behavior?

**DOI:** 10.3389/fncel.2013.00208

**Published:** 2013-11-15

**Authors:** Gianluca Serafini, Maurizio Pompili, Katelin F. Hansen, Karl Obrietan, Yogesh Dwivedi, Mario Amore, Noam Shomron, Paolo Girardi

**Affiliations:** ^1^Department of Neurosciences, Mental Health and Sensory Organs – Sant'Andrea Hospital, Sapienza University of RomeRome, Italy; ^2^Department of Neurosciences, Ohio State UniversityColumbus, OH, USA; ^3^Department of Psychiatry and Behavioral Neurobiology, University of Alabama at BirminghamBirmingham, AL, USA; ^4^Section of Psychiatry, Department of Neuroscience, Rehabilitation, Ophthalmology, Genetics, Maternal and Child Health University of GenovaGenova, Italy; ^5^Department of Cell and Developmental Biology, Sackler Faculty of Medicine, Tel Aviv UniversityTel Aviv, Israel

**Keywords:** microRNAs, synaptic plasticity, major affective disorders, suicidal behavior, gene expression

## Introduction

Major affective disorders are one of the foremost causes of morbidity worldwide; such disabling conditions are also frequently associated with suicidal behavior (Innamorati et al., 2011; Gonda et al., 2012; Serafini et al., 2012). Although many psychopharmacological agents are currently available, in particular for the treatment of major depressive disorder (MDD) (Serafini et al., 2013), our knowledge concerning the molecular and cellular mechanisms underlying this complex condition is still limited. Indeed, even minor alterations in the expression of genes regulating neural and structural plasticity may be crucial to understanding the pathogenesis of major affective disorders (Dwivedi et al., 2009a,b; Serafini et al., 2011, 2012).

MiRNAs are gene expression regulators critically affecting brain development that have been investigated as potential biomarkers for the diagnosis, management, treatment, and progression of neuropsychiatric disorders (Machado-Vieira et al., 2010; Saugstad, 2010; Dwivedi, 2011). Several facets of miRNA expression alterations are currently under investigation to gain insight into the pathology of neuronal disorders (Hansen et al., 2007; Lopez et al., 2013): miRNA expression alterations in pathophysiological models of disease (Ziu et al., 2011; Brandenburger et al., 2012); miRNA expression alterations in the blood of patients, most of which represent further downstream or compensatory effects (Schipper et al., 2007; Gallego et al., 2012); and miRNAs and their effectors acting as targets for the action of psychoactive drugs such as antidepressants and mood stabilizers (Zhou et al., 2009; Baudry et al., 2010; Oved et al., 2012).

Several miRNAs have emerged as potential mediators of depressive pathophysiology. The existence of polymorphisms in pre-miR-30e (Xu et al., 2010) and pre-miR-182 (Saus et al., 2010) has been associated with an increased risk of major depression. Depressive behavioral responses have been induced by miR-16 up-regulation in the raphe nuclei and hippocampus, with the latter associated with subsequent down-regulation of BNDF (Bai et al., 2012). MiR-16 down-regulation within the locus coeruleus was also induced in depressive mouse models (Launay et al., 2011). Smalheiser et al. (2012) investigated the expression of miRNAs in the prefrontal cortex (specifically in Brodmann Area 9) of 18 antidepressant-free depressed suicide victims, and 17 well-matched non-psychiatric controls, whose information was collected using the psychological autopsy method. In this study, global miRNA expression was significantly down-regulated by 17%, and 24 miRNAs were down-regulated by at least 30%. The authors also found significant down-regulation in an extensive inter-connected network of 21 miRNAs involved in cellular growth and differentiation. Notably, both the global decrease of miRNA expression, as well as its decreased variability, are consistent with hypo-activation of the frontal cortex in depressed subjects. Interestingly, the noted miRNAs have been suggested to be down-regulated in the frontal cortex of rats treated with corticosterone, and therefore, might be crucial in regulating stress-mediated miRNA expression in depressed subjects (Dwivedi et al., unpublished data). Hence, alterations in miRNA expression may be a fundamental event underlying gene network reorganization associated with major depression.

Nevertheless, a comprehensive understanding of miRNA networks dysregulated in major depression and induced by antidepressant medications as a function of brain region is currently unknown (Mouillet-Richard et al., 2012). It is also generally poorly understood how miRNA regulation affects cellular signaling networks in these biological processes. Here we provide an overview and critical review of the published work, particularly examining the role of miR-185 in major depression and suicidal behavior.

## The challenges of miRNA:mRNA target prediction in modeling pathology

Expression profiling and RNA sequencing of miRNAs have increased our understanding of which miRNAs are present in specific tissues, and how they may change under pathological conditions (Oved et al., 2012). However, once identified, linking a miRNA to its mRNA targets can be a challenging task, and the authenticity of functional miRNA:mRNA target pairs should be validated. A very small fraction of software-predicted miRNA targets are validated *in vivo*. As suggested by Kuppers et al. (2011), only a subset of predicted targets are consistently reduced in phenotypes that overexpress some miRNAs. Validation could be provided by showing a direct interaction between the miRNA and mRNA target. Further, miRNA silencing, overexpression, and luciferase reporter based assays are commonly used as supporting evidence for a functional interaction (Kuhn et al., 2008).

Many databases for miRNA target prediction have been created using an array of interrogation approaches. Considering varying degrees of sequence similarity, conservation, site accessibility, and variation in the targeted regions of the mRNA, the numerous databases can provide a surprising level of divergent results. This divergence speaks to the difficulty that scientists have had in developing a clear and concise set of rules underlying miRNA:mRNA interaction: clearly target prediction software should only be viewed as a tool to guide bench science.

Table 1 Provides a summary of current miRNA target prediction software that may be used to guide *in silico* investigations into miRNA and their putative targets. The analytical paradigms for each database are describes as well as advantages of each mode of inquiry.

**Table 1:** **Comprehensive list of miRNA target databases and software**.

**Databases for miRNAs and their target gene associations**	**Description**	**Release date of the last version**	**Web link**	**Publication**
DIANA–microT	This is an algorithm specifically trained on a positive and negative set of miRNA Recognition Elements (MREs) located in both the 3′-UTR and CDS regions. This new server detects miRNA targets in mRNA sequences of *Mus musculus, Drosophila melanogaster, C.elegans*, and *Homo sapiens*. It also provides hyperlinks to on-line servers such as iHOP and expression data for the selected miRNAs in tissues and cell lines. Each gene is weighted considering conserved as well as non-conserved sites. Furthermore, a signal to noise ratio is applied for each interaction in order to estimate the number of false positives.	Jul, 2012 (version 5.0)	http://www.microrna.gr/microT-CDS	Reczko et al. (2012)
MicroCosm	This is a miRNA target database that has been developed by Enright lab at EMBL-EBI. MicroCosm Targets includes computationally predicted microRNA targets for many species and is based on a dynamic program to search for maximal local complementarity alignments. The miRNA sequences are derived by the miRBase Sequence database and most genomic sequence from EnsEMBL. MicroCosm uses the miRanda algorithm that is one of the first miRNA target prediction algorithms.	Ago, 2010 (version v5)	http://www.ebi.ac.uk/enright-srv/microcosm/htdocs/targets/v5/	Griffiths-Jones et al. (2008)
miRanda and microRNA.org	It includes both software (miRanda) and a miRNA database (microRNA.org). This algorithm uses a score to rank the predictions according to a weighted sum based on matches, mismatches, and G:U wobbles. The newest version of MiRanda is also called mirSVR, it also considers a conservation measure based on the PhastCons conservation score.	Ago, 2010	http://www.microrna.org/microrna/home.do	Betel et al. (2008)
miRDB	All the targets are predicted by the bioinformatics tool MirTarget2, which analyzes thousands of genes impacted by miRNAs with an SVM learning machine. MiRDB includes predicted miRNA targets from the following organisms: humans, mouse, rat, dog, and chicken. MiRNA targets are predicted using common features associated with miRNA target binding. The positive and negative datasets for training consist of many negative and positive interactions, respectively.	Jan, 2012 (version 4.0)	http://mirdb.org/miRDB/	Wang and El Naqa (2008)
miRecords	This is an integrated microRNA target database for miRNA–target interactions. It provides miRNA-target relationships predicted by multiple target algorithms, such as Targetscan, PicTar, miRanda, PITA, and RNA22. It provides a large manually curated database including experimentally validated miRNA–target interactions with systematic documentation of experimental support for each interaction. It is expected to serve for experimental miRNA researchers, and informatics scientists.	Apr, 2013	http://mirecords.umn.edu/miRecords/	Xiao et al. (2009)
miRGator	This is an algorithm integrating the target prediction, functional analysis, gene expression data, and genome annotation. MiRNA function is inferred from the list of target genes predicted by other databases (miRanda, PicTar, and TargetScanS). For the expression analysis, miRGator integrates public expression findings of miRNAs with those of mRNAs and proteins.	Dic, 2012 (version 3.0)	http://genome.ewha.ac.kr/miRGator/miRGator.html	Nam et al. (2008)
miRGen++	miRGen is an integrated database including positional relationships between animal miRNAs and genomic annotation sets as well as animal miRNA targets, according to combinations of widely used target prediction programs. It provides comprehensive information about the position of human and mouse microRNA coding transcripts and their regulation by transcription factors.	Jan, 2007 (version 3)	http://www.diana.pcbi.upenn.edu/miRGen.html	Megraw et al. (2006)
MiRNAMap	This is an integrated database aimed to identify experimentally verified miRNA target genes in human, mouse, rat, and other metazoan genomes. MiRNA targets in 3′-UTR of genes and the miRNA targets were identified using the following computational tools: miRanda, RNAhybrid, and TargetScan. The putative miRNA targets are adequately filtered in order to reduce the false positive prediction rate of miRNA target sites.	Jul, 2007 (version 2.0)	http://mirnamap.mbc.nctu.edu.tw/	Hsu et al. (2008)
miRTarBase	This is an experimentally verified miRNA target base, including more than 3000 miRNA–target interactions collected by manually surveying pertinent literature. Generally, the collected miRNA–target interactions are validated experimentally based on reporter assays, western blot, or microarray experiments with overexpression or knockdown of miRNAs.	Jul, 2013 (version 4.3)	http://miRTarBase.mbc.nctu.edu.tw/	Hsu et al. (2011)
miRWalk	miRWalk is a comprehensive database providing information on miRNAs from human, and mouse, including their predicted and validated binding sites for their target genes. MiRWalk considers a newly developed algorithm aimed at creating the predicted miRNA binding sites on the complete sequences of all known genes of the human, mouse, and rat genomes. It provides predicted miRNA binding sites on genes associated with over 400 human biological pathways and over 2300 OMIM disorders. It also includes information on proteins known to be implicated in miRNA processing.	Mar, 2011	http://www.umm.uni-heidelberg.de/apps/zmf/mirwalk/	Dweep et al. (2011)
PicTar	PicTar is used for the identification and prediction of miRNA targets by combining multiple miRNAs or targets. This website provides details concerning: miRNA target predictions in vertebrates; miRNA target predictions in seven Drosophila species; miRNA targets in three nematode species; human miRNA targets that are not conserved but co-expressed (e.g., both the miRNA and mRNA that are expressed in the same tissue).	Mar, 2007	http://pictar.mdc-berlin.de/	Lall et al. (2006)
PITA	This algorithm predicts miRNA targets taking into account not only the specific duplex interaction information, but also the accessibility (the difference between the minimum free energy of the whole complex and the initial energy of a short mRNA region near the site) to the site in the mRNA. Several restrictions may be applied to reduce the set of resulting predictions.	Ago, 2008 (version 6)	http://genie.weizmann.ac.il/pubs/mir07/mir07_data.html	Kertesz et al. (2007)
RNA22	It is a pattern-based strategy aimed to identify the candidate targets. A Markov chain is used to identify those patters which are hypothesized to identify areas where the most statistically significant patterns map (target islands). The target islands are subsequently paired with miRNAs.	May, 2008 (version 1.1.2)	http://cbcsrv.watson.ibm.com/rna22.html	Miranda et al. (2006)
RNAhybrid	This algorithm provides a flexible software for predicting miRNA targets. It is a tool that finds the minimum free energy not only for short sequences as most of the mentioned algorithms, but also for the entire miRNA–mRNA. The analysis is carried out by hybridizing the short sequence to the best fitting part of the long sequence. Several restrictions like the number of unpaired bases and free energy allowed may be applied to reduce the set of resulting predictions.	Jul, 2013 (version 2.1.1–2)	http://bibiserv.techfak.uni-bielefeld.de/rnahybrid/	Kruger and Rehmsmeier (2006)
StarBase	This algorithm provides a public platform developed to facilitate the comprehensive exploration of miRNA–target interaction from CLIP-Seq (HITS-CLIP, PAR-CLIP) and degradome sequencing (PARE) data from the following organisms: human, mouse, *Caenhorhabditis elegans, Arabidopsis thaliana, Oryza sativa*, and *Vitis vinifera*. It also provides intersections of multiple target predictions, such as Targetscan, PicTar, miRanda, PITA, RNA22, and miRSVR.	Sep, 2011 (version 2.1)	http://starbase.sysu.edu.cn/	Yang et al. (2011)
TarBase	This is a large available target database including more than 65,000 miRNA-gene interactions. It includes targets derived from both microarrays and proteomics. It is linked to other databases such as Ensembl, Uniprot, RefSeq, and DIANA-microT enabling extension of each validated interaction with *in silico* predicted information.	Jan, 2009 (version 6.0)	http://microrna.gr/tarbase/	Vergoulis et al. (2012)
TargetRank	TargetRank scores the seed matches in a UTR relative to a given siRNA or miRNA and then calculates an overall score for the mRNA as a whole by summing the scores for all seed matches present in the 3′ UTR. Only targets with scores above 0.2 are reported. The relative ranking given by TargetRank may be considered more useful than the score itself.	Mar, 2006	http://genes.mit.edu/targetrank/	Nielsen et al. (2007)
TargetScan	This algorithm requires the seed complementary at least for 6 nt and considers the different seed types that have been defined using a specific hierarchy. It predicts microRNA targets from conserved UTR sequences by searching for the presence of conserved and non-conserved sites matching the seed region of each miRNA. In the last version of this algorithm, a multiple linear regression trained on 74 filtered datasets has been used to integrate determinants.	Mar, 2012 (version 6.1)	http://www.targetscan.org/	Friedman et al. (2009)

## MiR-185: A role in major affective disorders and suicidal behavior?

Preliminary studies have suggested the importance of miR-185 and miR-491-3p in the pathogenesis of major depression and suicidal behavior. MiR-185 is expressed in several brain regions such as the hippocampus and cortex, predominantly in synapses (Lugli et al., 2008; Xu et al., 2013). Earls et al. (2012) reported that miR-185 regulates cognitive and psychiatric symptoms of patients with the 22q11 deletion syndrome. Recently, Xu et al. (2013) suggested that miR185 controls the expression of Golgi-apparatus related genes including a new inhibitor of neuronal maturation. In particular, a reduction of miR-185 altered dendritic and spine development resulting in structural alterations of the hippocampus.

With respect to MDD and suicidality, miR-185 was shown to be upregulated in patients who completed suicide (Maussion et al., 2012). These increases in expression were correlated with reduced TrkB-T1, a truncated TrkB transcript whose downregulation has been associated with suicide (Ernst et al., 2009). The downregulation of TrkB-T1was associated with suicidal behavior in a sample of 38 suicide completers (60.5% having been previously diagnosed with MDD). Of note, five putative binding sites for miR-185 were found in the 3′ UTR of TrkB-T1 (using an *in silico* investigation). Array findings were confirmed with RT-PCR investigation and three of the five potential binding sites for miR-185 in the TrkB-T1 3′ UTR were demonstrated to be functional by luciferase assay. The authors did not find any confounding effect of age, pH, PMI, or suicide method. Through Pearson correlation and subsequent *in vitro* functional analyses (using silencing or exogenous expression of miR-185), TrkB-T1 levels and hsa-miR-185 levels were reported to be inversely correlated.

A few notes of caution should be mentioned with regard to the Maussion et al. (2012) study. The authors acknowledge that the underlying mechanism of increased miR-185 expression remains unclear. The study used HEK293 cells that yielded TrkB-T1 expression levels that were 10-fold greater than neuronal cell lines. Furthermore, RNA binding proteins, such as ELAVL1 or PABPC1, may be expressed in HEK293 cells (Drury et al., 2010) and potentially bind TrkB mRNA (Jain et al., 2011). Therefore, despite disproportionate increases in TrkB-T1 expression, the functional effect of hsa-miR-185 on TrkB-T1 observed in HEK293 cells might have been attenuated by the expression of these genes and their binding activity (George and Tenenbaum, 2006). Further, the study is limited by the small sample size and the negative findings in other brain regions, such as the cerebellum. Indeed, presumably only a limited part of the total variability in miRNAs that might regulate TrkB-T1 has been identified.

Of note, the subjects of the Maussion et al. (2012) study were not assessed for microduplications in the 22q11.2 region. This is of potential interest because the miR-185 locus maps to the 22q11.2 region, which has been associated with mood disorders such as depression and anxiety (Jolin et al., 2012; Weisfeld-Adams et al., 2012; Tang et al., 2013). Deletions of this region have also been consistently associated with schizophrenia (Karayiorgou et al., 2010) whereas duplications have been found in patients with autism (Lo-Castro et al., 2009). Alterations in the 22q11.2 region are also associated with morphological alterations in dendritic spines at glutamatergic synapses (Mukai et al., 2008), and abnormal maturation of miRNAs (Stark et al., 2008). Fénelon et al. (2013) have suggested that mice with a 22q11.2 microdeletion show significant alterations in high-frequency synaptic transmission, short- and long-term plasticity, and dendritic spine stability. The authors reported that variation in synaptic plasticity occurs by subtle changes in neuronal density and a reduction in inhibitory neuron. All of these alterations in neuronal function could play critical roles in depressive pathophysiology.

## Understanding the limitations of studies examining the role of miRNAs in major affective disorders

Since the first detection in *Caenorhabditis elegans* in 1993 (Lee et al., 1993), small interfering RNAs have raised great interest among neurobiologists for their potential role in neuropathological regulation. In line with this notion, large-scale analyses on post-mortem brains, as well as investigations in animal models of depression, have evaluated the impact of psychoactive medications on global miRNA expression. Transcriptome studies are now commonly used as a starting point to investigate the association between dysregulated miRNAs and major affective disorders. However, there are a number of conflicting studies with regard to the magnitude and direction of biologically-relevant miRNA expression changes in psychiatric disorders (Perkins et al., 2007; Beveridge et al., 2010). This could be due to tissue-specific variations in expression levels as well as heterogeneity in quantification and normalization procedures (Belzeaux et al., 2012). Furthermore, some studies on miRNAs and depression were conducted in peripheral blood despite uncertainties regarding how closely changes in peripheral miRNA expression reflect modifications in the central nervous system (e.g., Bocchio-Chiavetto et al., 2013). It is also worth noting that, “control” RNAs commonly used to normalize miRNA data (U6, U44, and U48) are very sensitive to post-mortem decay (Sadikovic et al., 2011) and thus, should be carefully matched among groups to prevent the emergence of artifactual shifts in miRNA expression. Finally, neuronal shrinkage, loss of glial cells, or loss of dendritic spines may also contribute to changes in miRNA levels. Clearly, changes in tissue composition or cellular compartments should be carefully taken into account when examining the available studies.

## Conclusion

Our understanding of the molecular mechanisms underlying major affective disorders may be significantly enriched by the knowledge of miRNAs' mechanisms of action. MiRNA targets are critically involved in stress-related disorders, neuroplasticity, and neurodevelopmental disorders (Rogaev, 2005). Given that miRNAs have been hypothesized to modulate ~50% of protein-coding genes and hundreds of mRNAs (Krol et al., 2010), a new level of complexity regarding gene expression has emerged. The entire miRNA context (both mRNA networks and their cellular environments) should be critically investigated when interpreting the effects of changes in miRNA levels. Much remains to be examined in order to translate these investigations into novel therapeutics for the treatment of psychiatric conditions.

## References

[B1] Bai M. Zhu X. Zhang Y. Zhang S. Zhang L. Xue L. (2012). Abnormal hippocampal bdnf and mir-16 expression is associated with depression-like behaviors induced by stress during early life. PLoS ONE 7:e46921 10.1371/journal.pone.0046921 23056528 PMC3466179

[B2] Baudry A. Mouillet-Richard S. Schneider B. Launay J. M. Kellermann O. (2010). miR-16 targets the serotonin transporter: a new facet for adaptive responses to antidepressants. Science 329, 1537–1541 10.1126/science.1193692 20847275

[B3] Belzeaux R. Bergon A. Jeanjean V. Loriod B. Formisano-Tréziny C. Verrier L. (2012). Responder and non-responder patients exhibit different peripheral transcriptional signatures during major depressive episode. Transl. Psychiatry 2, e185 10.1038/tp.2012.112 23149449 PMC3565773

[B4] Betel D. Wilson M. Gabow A. Marks D. S. Sander C. (2008). The microRNA. org resource. targets and expression. Nucl. Acids Res. 36, D149–D153 10.1093/nar/gkm995 18158296 PMC2238905

[B5] Beveridge N. J. Gardiner E. Carroll A. P. Tooney P. A. Cairns M. J. (2010). Schizophrenia is associated with an increase in cortical microRNA biogenesis. Mol. Psychiatry 15, 1176–1189 10.1038/mp.2009.84 19721432 PMC2990188

[B6] Bocchio-Chiavetto L. Maffioletti E. Bettinsoli P. Giovannini C. Bignotti S. Tardito D. (2013). Blood microRNA changes in depressed patients during antidepressant treatment. Eur. Neuropsychopharmacol. 23, 602–611 10.1016/j.euroneuro.2012.06.013 22925464

[B7] Brandenburger T. Castoldi M. Brendel M. Grievink H. Schlösser L. Werdehausen R. (2012). Expression of spinal cord microRNAs in a rat model of chronic neuropathic pain. Neurosci. Lett. 506, 281–286 10.1016/j.neulet.2011.11.023 22138088

[B8] Drury G. L. Di Marco S. Dormoy-Raclet V. Desbarats J. Gallouzi I. E. (2010). FasL expression in activated T lymphocytes involves HuR-mediated stabilization. J. Biol. Chem. 285, 31130–31138 10.1074/jbc.M110.137919 20675370 PMC2951186

[B9] Dweep H. Sticht C. Pandey P. Gretz N. (2011). miRWalk–database: prediction of possible miRNA binding sites by walking the genes of 3 genomes. J. Biomed. Inform. 44, 839–837 10.1016/j.jbi.2011.05.002 21605702

[B10] Dwivedi Y. (2011). Evidence demonstrating role of microRNAs in the etiopathology of major depression. J Chem Neuroanat 42, 142–156 10.1016/j.jchemneu.2011.04.002 21515361 PMC3163781

[B11] Dwivedi Y. Rizavi H. Zhang H. Mondal A. C. Roberts R. C. Conley R. R. (2009a). Neurotrophin receptor activation and expression in human postmortem brain: effect of suicide. Biol. Psychiatry 65, 319–328 10.1016/j.biopsych.2008.08.035 18930453 PMC2654767

[B12] Dwivedi Y. Rizavi H. S. Zhang H. Roberts R. C. Conley R. R. Pandey G. N. (2009b). Aberrant extracellular signal-regulated kinase (ERK)1/2 signaling in suicide brain: role of ERK kinase 1 (MEK1). Int. J. Neuropsychopharmacol. 12, 1337–1354 10.1017/S1461145709990575 19835659

[B13] Earls L. R. Fricke R. G. Yu J. Berry R. B. Baldwin L. T. Zakharenko S. S. (2012). Age-dependent microRNA control of synaptic plasticity in 22q11 deletion syndrome and schizophrenia. J. Neurosci. 32, 14132–14144 10.1523/JNEUROSCI.1312-12.2012 23055483 PMC3486522

[B14] Ernst C. Deleva V. Deng X. Sequeira A. Pomarenski A. Klempan T. (2009). Alternative splicing, methylation state, and expression profile of tropomyosin-related kinase B in the frontal cortex of suicide completers. Arch. Gen. Psychiatry 66, 22–32 10.1001/archpsyc.66.1.22 19124685

[B15] Fénelon K. Xu B. Lai C. S. Mukai J. Markx S. Stark K. L. (2013). The pattern of cortical dysfunction in a mouse model of a schizophrenia-related microdeletion. J. Neurosci. 33, 14825–14839 10.1523/JNEUROSCI.1611-13.2013 24027283 PMC3771024

[B16] Friedman R. C. Farh K. K. Burge C. B. Bartel D. P. (2009). Most mammalian mRNAs are conserved targets of microRNAs. Genome Res. 19, 92–105 10.1101/gr.082701.108 18955434 PMC2612969

[B17] Gallego J. A. Gordon M. L. Claycomb K. Bhatt M. Lencz T. Malhotra A. K. (2012). *In vivo* microRNA detection and quantitation in cerebrospinal fluid. J. Mol. Neurosci. 47, 243–248 10.1007/s12031-012-9731-7 22402993 PMC3361591

[B18] George A. D. Tenenbaum S. A. (2006). MicroRNA modulation of RNA-binding protein regulatory elements. RNA Biol. 3, 57–59 10.4161/rna.3.2.3250 17114943

[B19] Gonda X. Pompili M. Serafini G. Montebovi F. Campi S. Dome P. (2012). Suicidal behavior in bipolar disorder: epidemiology, characteristics and major risk factors. J. Affect. Disord. 143, 16–26 10.1016/j.jad.2012.04.041 22763038

[B20] Griffiths-Jones S. Saini H. K. van Dongen S. Enright A. J. (2008). miRBase: tools for microRNA genomics. Nucl. Acids Res. 36, D154–D158 10.1093/nar/gkm952 17991681 PMC2238936

[B21] Hansen T. Olsen L. Lindow M. Jakobsen K. D. Ullum H. Jonsson E. (2007). Brain expressed microRNAs implicated in schizophrenia etiology. PLoS ONE 2:e873 10.1371/journal.pone.0000873 17849003 PMC1964806

[B22] Hsu S. D. Chu C. H. Tsou A. P. Chen S. J. Chen H. C. Hsu P. W. (2008). miRNAMap 2.0: genomic maps of microRNAs in metazoan genomes. Nucl. Acids Res. 36, D165–D169 10.1093/nar/gkm1012 18029362 PMC2238982

[B23] Hsu S. D. Lin F. M. Wu W. Y. Liang C. Huang W. C. Chan W. L. (2011). miRTarBase: a database curates experimentally validated microRNA-target interactions. Nucl. Acids Res. 39, D163–169 10.1093/nar/gkq1107 21071411 PMC3013699

[B24] Innamorati M. Pompili M. Gonda X. Amore M. Serafini G. Niolu C. (2011). Psychometric properties of the gotland scale for depression in Italian psychiatric inpatients and its utility in the prediction of suicide risk. J. Affect. Disord. 132, 99–103 10.1016/j.jad.2011.02.003 21371756

[B25] Jain R. Devine T. George A. D. Chittur S. V. Baroni T. E. Penalva L. O. (2011). RIP-chip analysis: RNA-binding protein immunoprecipitation-microarray (chip) profiling. Methods Mol. Biol. 703, 247–263 10.1007/978-1-59745-248-9_17 21125495

[B26] Jolin E. M. Weller R. A. Weller E. B. (2012). Occurrence of affective disorders compared to other psychiatric disorders in children and adolescents with 22q11.2 deletion syndrome. J. Affect. Disord. 136, 222–228 10.1016/j.jad.2010.11.025 21215459

[B27] Karayiorgou M. Simon T. J. Gogos J. A. (2010). 22q11.2 microdeletions: linking DNA structural variation to brain dysfunction and schizophrenia. Nat. Rev. Neurosci. 11, 402–416 10.1038/nrn2841 20485365 PMC2977984

[B28] Kertesz M. Iovino N. Unnerstall U. Gaul U. Segal E. (2007). The role of site accessibility in microRNA target recognition. Nat. Genet. 39, 1278–1284 10.1038/ng2135 17893677

[B29] Krol J. Loedige I. Filipowicz W. (2010). The widespread regulation of microRNA bio-genesis, function and decay. Nat. Rev. Genet. 11, 597–610 10.1038/nrg2843 20661255

[B30] Kruger J. Rehmsmeier M. (2006). RNAhybrid: microRNA target prediction easy, fast and flexible. Nucl. Acids Res. 34, W451–W454 10.1093/nar/gkl243 16845047 PMC1538877

[B31] Kuhn D. E. Martin M. M. Feldman D. S. Terry A. V. Jr. Nuovo G. J. Elton T. S. (2008). Experimental validation of miRNA targets. Methods 44, 47–54 10.1016/j.ymeth.2007.09.005 18158132 PMC2237914

[B32] Kuppers D. A. Hwang H. C. Jackson A. L. Linsley P. S. Clurman B. E. Fero M. L. (2011). Effect of Xpcl1 activation and p27(Kip1) loss on gene expression in murine lymphoma. PLoS ONE 6:e14758 10.1371/journal.pone.0014758 21412408 PMC3055866

[B33] Lall S. Grün D. Krek A. Chen K. Wang Y. L. L. Dewey C. N. N. (2006). A genome-wide map of conserved microRNA targets in *C. elegans*. Curr. Biol. 16, 460–471 10.1016/j.cub.2006.01.050 16458514

[B34] Launay J. M. Mouillet-Richard S. Baudry A. Pietri M. Kellermann O. (2011). Raphe-mediated signals control the hippocampal response to SRI antidepressants via miR-16. Transl. Psychiatry 1, e56 10.1038/tp.2011.54 22833211 PMC3309472

[B35] Lee R. C. Feinbaum R. L. Ambros V. (1993). The *C. elegans* heterochronic gene lin-4 encodes small RNAs with antisense complementarity to lin-14. Cell 75, 843–854 10.1016/0092-8674(93)90529-Y 8252621

[B36] Lo-Castro A. Galasso C. Cerminara C. El-Malhany N. Benedetti S. Nardone A. M. (2009). Association of sindromi mental retardation and autismwith 22q11.2 duplication. Neuropediatrics 40, 137–140 10.1055/s-0029-1237724 20020400

[B37] Lopez J. P. Fiori L. M. Gross J. A. Labonte B. Yerko V. Mechawar N. (2013). Regulatory role of miRNAs in polyamine gene expression in the prefrontal cortex of depressed suicide completers. Int. J. Neuropsychopharmacol. 12, 1–10 10.1017/S1461145713000941 24025154

[B38] Lugli G. Torvik V. I. Larson J. Smalheiser N. R. (2008). Expression of microRNAs and their precursors in synaptic fractions of adult mouse forebrain. J. Neurochem. 106, 650–661 10.1111/j.1471-4159.2008.05413.x 18410515 PMC3711666

[B39] Machado-Vieira R. Salvadore G. DiazGranados N. Ibrahim L. Latov D. Wheeler-Castillo C. (2010). New therapeutic targets for mood disorders. Sci. World. J. 10, 713–726 10.1100/tsw.2010.65 20419280 PMC3035047

[B40] Maussion G. Yang J. Yerko V. Barker P. Mechawar N. Ernst C. (2012). Regulation of a truncated form of tropomyosin-related kinase B (TrkB) by Hsa-miR-185^*^ in frontal cortex of suicide completers. PLoS ONE 7:e39301 10.1371/journal.pone.0039301 22802923 PMC3382618

[B41] Megraw M. Sethupathy P. Corda B. Hatzigeorgiou A. G. (2006). miRGen: a database for the study of animal microRNA genomic organization and function. Nucl. Acids Res. 35, D149–D155 10.1093/nar/gkl904 17108354 PMC1669779

[B42] Miranda K. C. Huynh T. Tay Y. Ang Y. S. S. Tam W. L. L. Thomson A. M. (2006). A pattern-based method for the identification of microRNA binding sites and their corresponding heteroduplexes. Cell 126, 1203–1217 10.1016/j.cell.2006.07.031 16990141

[B43] Mouillet-Richard S. Baudry A. Launay J. M. Kellermann O. (2012). MicroRNAs and depression. Neurobiol. Dis. 46, 272–278 10.1016/j.nbd.2011.12.035 22226785

[B44] Mukai J. Dhilla A. Drew L. J. Stark K. L. Cao L. MacDermott A. B. (2008). Palmitoylationdependent neurodevelopmental deficits in a mouse model of 22q11 microdeletion. Nat. Neurosci. 11, 1302–1310 10.1038/nn.2204 18836441 PMC2756760

[B45] Nam S. Kim B. Shin S. Lee S. (2008). miRGator: an integrated system for functional annotation of microRNAs. Nucl. Acids Res. 36, 159–164 10.1093/nar/gkm829 17942429 PMC2238850

[B46] Nielsen C. B. Shomron N. Sandberg R. Hornstein E. Kitzman J. Burge C. B. (2007). Determinants of targeting by endogenous and exogenous microRNAs and siRNAs. RNA 13, 1894–1910 10.1261/rna.768207 17872505 PMC2040081

[B47] Oved K. Morag A. Pasmanik-Chor M. Oron-Karni V. Shomron N. Rehavi M. (2012). Genome-wide miRNA expression profiling of human lymphoblastoid cell lines identifies tentative SSRI antidepressant response biomarkers. Pharmacogenomics 13, 1129–1139 10.2217/pgs.12.93 22909203

[B48] Perkins D. O. Jeffries C. D. Jarskog L. F. Thomson J. M. Woods K. Newman M. A. (2007). microRNA expression in the prefrontal cortex of individuals with schizophrenia and schizoaffective disorder. Genome Biol. 8, R27 10.1186/gb-2007-8-2-r27 17326821 PMC1852419

[B49] Reczko M. Maragkakis M. Alexiou P. Grosse I. Hatzigeorgiou A. G. (2012). Functional microRNA targets in protein coding sequences. Bioinformatics 28, 771–776 10.1093/bioinformatics/bts043 22285563

[B50] Rogaev E. I. (2005). Small RNAs in human brain development and disorders. Biochemistry (Mosc) 70, 1404–1407 10.1007/s10541-005-0276-z 16417465

[B51] Sadikovic B. Pearson R. Meng L. Beaudet A. (2011). Identification of miRNAs and target mRNAs with degulated expression in schizophrenia and bipolar brains, in 12th International Congress of Human Genetics, (Montreal), 586T, 219

[B52] Saugstad J. A. (2010). MicroRNAs as effectors of brain function with roles in ischemia and injury, neuroprotection, and neurodegeneration. J. Cereb. Blood Flow. Metab. 30, 1564–1576 10.1038/jcbfm.2010.101 20606686 PMC2932764

[B53] Saus E. Soria V. Escaramis G. Vivarelli F. Crespo J. M. Kagerbauer B. (2010). Genetic variants and abnormal processing of pre-miR-182, a circadian clock modulator, in major depression patients with late insomnia. Hum. Mol. Genet. 19, 4017–4025 10.1093/hmg/ddq316 20656788

[B54] Schipper H. M. Maes O. C. Chertkow H. M. Wang E. (2007). MicroRNA expression in Alzheimer blood mononuclear cells. Gene Regul. Syst. Bio. 1, 263–274 19936094 10.4137/grsb.s361 PMC2759133

[B55] Serafini G. Pompili M. Innamorati M. Dwivedi Y. Brahmachari G. Girardi P. (2013). Pharmacological properties of glutamatergic drugs targeting NMDA receptors and their application in major depression. Curr. Pharm. Des. 19, 1898–1922 10.2174/13816128113199990293 23173582

[B56] Serafini G. Pompili M. Innamorati M. Giordano G. Montebovi F. Sher L. (2012). The role of microRNAs in synaptic plasticity, major affective disorders and suicidal behavior. Neurosci. Res. 73, 179–190 10.1016/j.neures.2012.04.001 22521503

[B57] Serafini G. Pompili M. Innamorati M. Giordano G. Tatarelli R. Lester D. (2011). Glycosides, depression and suicidal behaviour: the role of glycoside-linked proteins. Molecules 16, 2688–2713 10.3390/molecules16032688 21441870 PMC6259655

[B58] Smalheiser N. R. Lugli G. Rizavi H. S. Torvik V. I. Turecki G. Dwivedi Y. (2012). MicroRNA expression is down-regulated and reorganized in prefrontal cortex of depressed suicide subjects. PLoS ONE 7:e33201 10.1371/journal.pone.0033201 22427989 PMC3302855

[B59] Stark K. L. Xu B. Bagchi A. Lai W. S. Liu H. Hsu R. (2008). Altered brain microRNA biogenesis contributes to phenotypic deficits in a 22q11-deletion mouse model. Nat. Genet. 40, 751–760 10.1038/ng.138 18469815

[B60] Tang S. X. Yi J. J. Calkins M. E. Whinna D. A. Kohler C. G. Souders M. C. (2013). Psychiatric disorders in 22q11.2 deletion syndrome are prevalent but undertreated. Psychol. Med. 9, 1–11 10.1017/S0033291713001669 24016317 PMC4461220

[B61] Vergoulis T. I. Vlachos P. Alexiou G. Georgakilas M. Maragkakis M. Reczko S. (2012). Tarbase 6.0: capturing the exponential growth of miRNA targets with experimental support. Nucl. Acids Res. 40, D222–D229 10.1093/nar/gkr1161 22135297 PMC3245116

[B62] Wang X. El Naqa I. M. (2008). Prediction of both conserved and non-conserved microRNA targets in animals. Bioinformatics 24, 325–332 10.1093/bioinformatics/btm595 18048393

[B63] Weisfeld-Adams J. D. Edelmann L. Gadi I. K. Mehta L. (2012). Phenotypic heterogeneity in a family with a small atypical microduplication of chromosome 22q11.2 involving TBX1. Eur. J. Med. Genet. 55, 732–736 10.1016/j.ejmg.2012.08.011 23059467

[B64] Xiao F. Zuo Z. Cai G. Kang S. Gao X. Li T. (2009). miRecords: an integrated resource for microRNA-target interactions. Nucl. Acids Res. 37, D105–D110 10.1093/nar/gkn851 18996891 PMC2686554

[B65] Xu B. Hsu P. K. Stark K. L. Karayiorgou M. Gogos J. A. (2013). Derepression of a neuronal inhibitor due to miRNA dysregulation in a schizophrenia-related microdeletion. Cell 152, 262–275 10.1016/j.cell.2012.11.052 23332760 PMC3556818

[B66] Xu Y. Liu H. Li F. Sun N. Ren Y. Liu Z. (2010). A polymorphism in the microRNA-30e precursor associated with major depressive disorder risk and P300 waveform. J. Affect. Disord. 127, 332–336 10.1016/j.jad.2010.05.019 20579744

[B67] Yang J. H. Li J. H. Shao P. Zhou H. Chen Y. Q. Qu L. H. (2011). starBase: a database for exploring microRNA-mRNA interaction maps from Argonaute CLIP-Seq and Degradome-Seq data. Nucl. Acids Res. 39, D202–D209 10.1093/nar/gkq1056 21037263 PMC3013664

[B68] Zhou R. Yuan P. Wang Y. Hunsberger J. G. Elkahloun A. Wei Y. (2009). Evidence for selective microRNAs and their effectors as common long-term targets for the actions of mood stabilizers. Neuropsychopharmacology 34, 1395–1405 10.1038/npp.2008.131 18704095 PMC2669666

[B69] Ziu M. Fletcher L. Rana S. Jimenez D. F. Digicaylioglu M. (2011). Temporal differences in microRNA expression patterns in astrocytes and neurons after ischemic injury. PLoS ONE 6:e14724 10.1371/journal.pone.0014724 21373187 PMC3044134

